# Impressive Skin and Systemic Response Despite Patient Underestimation and Delayed Diagnosis of Blastic Plasmacytoid Dendritic Cell Neoplasm

**DOI:** 10.1155/crh/4591883

**Published:** 2026-07-18

**Authors:** Emanuele Pacini, Beatrice Esposito Vangone, Adele Santoni, Margherita Malchiodi, Corrado Zuanelli Brambilla, Anna Sicuranza, Giulia Beffa, Monica Bocchia

**Affiliations:** ^1^ Hematology Unit, University Hospital of Siena, Siena, Italy, unisi.it; ^2^ Hematology Unit, San Donato Hospital, Arezzo, Italy, sandonato-gsd.it; ^3^ Stem Cell Transplant and Cellular Therapy Unit, University Hospital of Siena, Siena, Italy, unisi.it

## Abstract

Blastic plasmacytoid dendritic cell neoplasm (BPDCN) is a rare and aggressive hematologic malignancy derived from precursors of plasmacytoid dendritic cells (pDCs), most frequently presenting with cutaneous involvement and often progressing to bone marrow (BM), lymph node, and central nervous system (CNS) infiltration. Owing to its heterogeneous and often indolent initial presentation, diagnosis is frequently delayed, potentially worsening prognosis. We report the case of a 55‐year‐old man with long‐standing underrecognized cutaneous and nodal lesions that eventually evolved into disseminated BPDCN. The patient was successfully treated with intensive chemotherapy (CHT) using a hyper‐CVAD regimen combined with intrathecal therapy (IT), achieving complete remission (CR). Consolidation with allogeneic hematopoietic stem cell transplantation (allo‐HSCT) from a matched sibling donor resulted in sustained minimal residual disease (MRD)–negative CR. At 23 months post transplant, the patient remains disease‐free with full donor chimerism and no evidence of graft‐versus‐host disease (GvHD). This case highlights the diagnostic challenges of BPDCN, the importance of early recognition of atypical cutaneous lesions, and supports the role of intensive CHT followed by allo‐HSCT as an effective therapeutic strategy in eligible patients.

## 1. Introduction

Blastic plasmacytoid dendritic cell neoplasm (BPDCN) is a rare and aggressive hematologic malignancy originating from precursors of plasmacytoid dendritic cells (pDCs), accounting for less than 1% of all acute leukemias and myeloid neoplasms. According to the fifth edition of the World Health Organization Classification of Hematolymphoid Tumors, BPDCN is classified among myeloid and dendritic cell neoplasms and is defined by a characteristic immunophenotype rather than a specific genetic alteration [1]. Clinically, BPDCN most commonly presents with skin involvement, observed in up to 90% of cases at diagnosis, often as bruise‐like patches, nodules, or plaques that may be misinterpreted as benign dermatologic conditions. Disease progression frequently includes bone marrow (BM), lymph node, splenic, and central nervous system (CNS) involvement, either at diagnosis or during relapse [2, 3]. Leukemic presentation, although less frequent, is associated with aggressive clinical behavior and poor outcomes [4]. The diagnosis of BPDCN relies on integrated morphologic, immunophenotypic, and clinical assessment. Neoplastic cells typically express CD4, CD123, HLA‐DR, TCL1, and frequently CD56, while lacking lineage‐specific markers of myeloid, B‐ or T‐cell differentiation [3, 5]. Despite advances in diagnostic strategies, BPDCN remains challenging to recognize, particularly in patients with slowly progressive or isolated cutaneous disease, leading to diagnostic delays [6]. Therapeutically, BPDCN has historically been associated with poor prognosis when treated with conventional chemotherapy (CHT) alone. Recent advances, including CD123‐targeted therapies and novel combination strategies such as triplet regimens with tagraxofusp, azacitidine, and venetoclax, have improved outcomes in BPDCN. The activity of venetoclax‐based approaches is consistent with the recognized BCL2 dependence of the disease [7, 8]; however, access to these agents may be limited, and CNS involvement remains challenging due to the need for integrated CNS‐directed therapy [2, 9]. In this context, intensive acute lymphoblastic leukemia–like regimens, such as hyper‐CVAD, followed by consolidation with allogeneic hematopoietic stem cell transplantation (allo‐HSCT) in the first complete remission (CR), remain a cornerstone of treatment for fit patients [9]. Here, we report a case of BPDCN, highlighting the diagnostic challenges posed by indolent cutaneous presentation and the role of intensive multimodal therapy in achieving durable disease control.

## 2. Case Presentation

**FIGURE 1:**
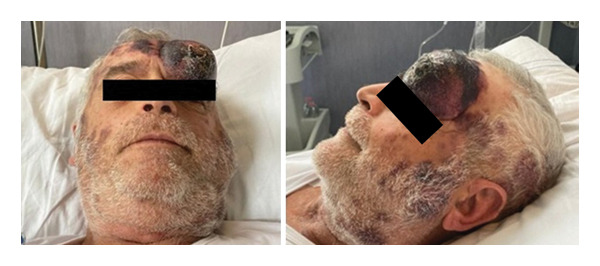
Patient’s lesions at diagnosis.

**FIGURE 2:**
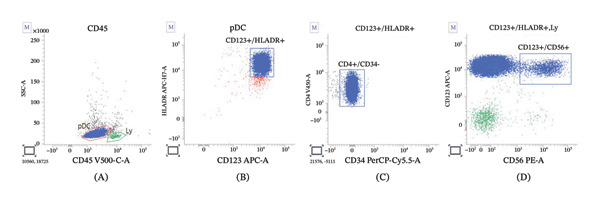
Immunophenotypic analysis of the cerebrospinal fluid. (A) CD45 vs SSC: blastic plasmacytoid dendritic cells (blue) show lower CD45 expression than lymphocytes (green) and display uniform expression of CD123 and HLA‐DR (B). In addition, blastic plasmacytoid dendritic cells are CD4+ and CD34‐‐ (C) and partially express the CD56 antigen (D).

**FIGURE 3:**
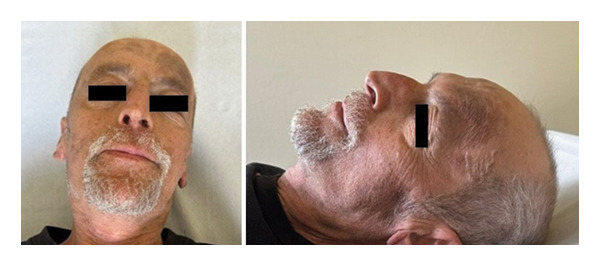
Patient’s complete resolution of skin lesions after treatment.

We report the case of a 55‐year‐old man with extensive, initially underrecognized cutaneous and nodal lesions that were ultimately diagnosed as BPDCN at the Hematology Unit of Azienda Ospedaliera Universitaria Senese. The patient first noticed a subtle enlargement of the left parotid gland and a slowly expanding, nonpainful mass over the left eyebrow. Despite the persistent and slow progression of these lesions, initially interpreted as hematoma, he did not seek medical advice until 18 months later, when multiple skin nodules and diffuse lymphadenopathies also appeared (Figure 1). A biopsy of a cutaneous nodule and parotid mass was performed, revealing a lymphoid infiltrate composed of medium‐to large‐sized cells with blastic morphology and a typical BPDCN immunohistochemical phenotype. Whole‐body contrast‐enhanced CT scan and 18‐FDG positron emission tomography confirmed pathological enhancement of the subcutaneous forehead mass and demonstrated additional involvement of the chest, back, abdominal skin, both parotid glands, nasopharynx, tonsils, maxilla, and diffuse superficial lymph nodes. In BM, a minimal involvement by BPDCN cells (1% of total cellularity) was detected. Cytogenetic evaluation of BM aspirate documented a normal male karyotype (46, XY), and molecular analysis did not identify NPM1 or FLT3 mutations; next‐generation sequencing was not performed. BPDCN cells (CD123+, HLA‐DR+, CD4+, CD56+, CD38+, and CD34‐) were also identified in the cerebrospinal fluid (CSF) (Figure 2). At admission to our unit, the patient was in good clinical condition. Full blood count showed white blood cells 5,8 × 10^3^/μL, hemoglobin 13,4 g/dL, and platelets 248 × 10^3^/μL. Blood chemistry was within normal ranges, except for mildly increased lactate dehydrogenase levels (326 UI/L). Since tagraxofusp was not registered in Italy for BPDCN with CNS involvement, the patient was started on a hyper‐CVAD CHT regimen [6] combined with intrathecal therapy (IT) (methotrexate 12.5 mg, cytarabine 50 mg, and dexamethasone 4 mg), administered four times during the first cycle (with CSF clearance after the third dose) and once during the second cycle. After 2 cycles of intravenous and intrathecal CHT, he achieved a complete clinical resolution of skin lesions and all disease localizations, including CSF clearance; immunophenotypic analysis of a BM aspirate showed 0.46% minimal residual disease (MRD). Following a third hyper‐CVAD course (the planned intrathecal administration was omitted due to a bilateral frontal subdural hematoma), the patient underwent allo‐HSCT from a fully matched sibling donor after myeloablative conditioning with thiotepa, busulfan, and fludarabine. Graft‐versus‐host disease (GvHD) prophylaxis consisted of cyclosporine A, methotrexate, and antithymocyte globulin. At 23 months after allo‐HSCT, the patient remains MRD‐negative in CR with full donor chimerism and no evidence of relapse or GvHD (Figure 3).

## 3. Discussion

BPDCN is a clinically and biologically aggressive malignancy characterized by a wide spectrum of presentations, which often complicates and delays diagnosis. Cutaneous lesions are typically the earliest manifestation and may precede systemic dissemination by months or years, as observed in our patient. The prolonged period of indolent skin and nodal involvement, initially misinterpreted as benign lesions, exemplifies one of the major diagnostic pitfalls of BPDCN and is consistent with previously reported cases [3, 6]. Despite the extensive extramedullary disease burden at presentation, BM involvement in our patient was minimal, highlighting the dissociation that can occur between peripheral disease extent and marrow infiltration. Importantly, CNS involvement was detected early through cerebrospinal fluid analysis, reinforcing current recommendations to systematically evaluate CNS disease in BPDCN, even in the absence of neurological symptoms [3, 10]. From a therapeutic perspective, the optimal management of BPDCN remains a subject of ongoing investigation. While CD123‐targeted therapy with tagraxofusp has demonstrated high response rates, particularly in treatment‐naïve patients, its availability is limited in some countries, and data on CNS penetration and efficacy remain limited [2]. In this setting, intensive CHT regimens traditionally used in acute lymphoblastic leukemia, such as hyper‐CVAD, represent a valid alternative, especially for patients with CNS involvement [9]. Our patient achieved a rapid and deep response after two cycles of hyper‐CVAD combined with IT, supporting the effectiveness of this approach in achieving disease control across multiple compartments. Allo‐HSCT in first CR is widely considered the most effective consolidative strategy for eligible BPDCN patients, offering the best chance for long‐term survival through a graft‐versus‐leukemia effect [2, 9]. In our case, consolidation with allo‐HSCT from a matched sibling donor resulted in sustained MRD‐negative remission, full donor chimerism, and absence of GvHD at nearly 2 years of follow‐up. This favorable outcome aligns with published data, indicating improved progression‐free and overall survival in patients undergoing allo‐HSCT during first remission [9]. In conclusion, this case underscores several key aspects of BPDCN management: the need for heightened clinical suspicion in patients with atypical, slowly progressive cutaneous lesions; the importance of comprehensive staging, including CNS evaluation; and the effectiveness of intensive CHT followed by allo‐HSCT in achieving durable remission. Early diagnosis and prompt referral to specialized hematologic centers remain critical to improving outcomes in this rare and challenging disease.

## Funding

No funding was received for this study.

## Disclosure

All authors have read and approved the final version of the manuscript. Anna Sicuranza (corresponding author) had full access to all of the data in this study and takes complete responsibility for the integrity of the data and the accuracy of the data analysis.

## Consent

Written informed consent was obtained from the patient for publication of this case report and the accompanying clinical images.

## Conflicts of Interest

The authors declare no conflicts of interest.

## Supporting Information

Additional supporting information can be found online in the Supporting Information section.

## Supporting information


**Supporting Information** CARE‐checklist case report.

## Data Availability

Data sharing is not applicable to this article as no datasets were generated or analyzed during the current study.
