# Supplementary material for: Impressive Skin and Systemic Response Despite Patient Underestimation and Delayed Diagnosis of Blastic Plasmacytoid Dendritic Cell Neoplasm
Source: Case Rep Hematol. 2026 Jul 18;2026:4591883. doi: 10.1155/crh/4591883 (PMC13379766; doi:10.1155/crh/4591883)
Supplement: Supplementary file 1 — Supporting Information CARE‐checklist case report. [file CRH-2026-4591883-s001.pdf]

CARE case report guidelines

CARE Checklist of information to include when writing a case report

(cc) BY-NC-ND

Topic	Item	Checklist item description	Reported on Line
Title	1	The diagnosis or intervention of primary focus followed by the words "case report"	BPDCN
Key Words	2	2 to 5 key words that identify diagnoses or interventions in this case report, including "case report"	BPDCN; case report, allo-HCT
Abstract (no references)	3a	Introduction: What is unique about this case and what does it add to the scientific literature?	successful treatment outcome in a rare disease
	3b	Main symptoms and/or important clinical findings	Parotid enlargement, eyebrow mass, skin nodules, lymphadenopathies
	3c	The main diagnoses, therapeutic interventions, and outcomes	BPDCN treated with Hyper-CVAD, IT therapy, and allo-HSCT
	3d	Conclusion—What is the main "take-away" lesson(s) from this case?	Early recognition and intensive treatment may improve outcomes
Introduction	4	One or two paragraphs summarizing why this case is unique (**may include references**)	Diagnostic challenges and treatment strategies of BPDCN reviewed
Patient Information	5a	De-identified patient specific information.	55-year-old male
	5b	Primary concerns and symptoms of the patient.	Progressive cutaneous lesions and parotid swelling
	5c	Medical, family, and psycho-social history including relevant genetic information	Unremarkable; no relevant history reported
	5d	Relevant past interventions with outcomes	No previous specific treatment before diagnosis
Clinical Findings	6	Describe significant physical examination (PE) and important clinical findings.	Multiple skin lesions, parotid involvement, diffuse lymphadenopathies
Timeline	7	Historical and current information from this episode of care organized as a timeline	Described in the text of the case presentation
Diagnostic Assessment	8a	Diagnostic testing (such as PE, laboratory testing, imaging, surveys).	Biopsy, PET/CT, BM evaluation, CSF, flow cytometry and cytogenetics analysis
	8b	Diagnostic challenges (such as access to testing, financial, or cultural)	Indolent presentation and delay in reporting doctors
	8c	Diagnosis (including other diagnoses considered)	BPDCN with skin, lymph node, BM, and CNS involvement
	8d	Prognosis (such as staging in oncology) where applicable	Poor prognosis
Therapeutic Intervention	9a	Types of therapeutic intervention (such as pharmacologic, surgical, preventive, self-care)	Chemotherapy, intrathecal therapy, allo-HSCT
	9b	Administration of therapeutic intervention (such as dosage, strength, duration)	Three Hyper-CVAD cycles plus intrathecal methotrexate/cytarabine/dexamethasone
	9c	Changes in therapeutic intervention (with rationale)	Intrathecal therapy omitted during cycle 3 due to subdural hematoma
Follow-up and Outcomes	10a	Clinician and patient-assessed outcomes (if available)	Complete clinical and hematologic remission
	10b	Important follow-up diagnostic and other test results	MRD-negative BM, full donor chimerism, no relapse
	10c	Intervention adherence and tolerability (How was this assessed?)	Treatment was well tolerated both clinically and biochemically
	10d	Adverse and unanticipated events	Bilateral frontal subdural hematoma
Discussion	11a	A scientific discussion of the strengths AND limitations associated with this case report	Long follow-up; NGSg was not performed
	11b	Discussion of the relevant medical literature **with references**.	Current evidence on BPDCN diagnosis and treatment reviewed
	11c	The scientific rationale for any conclusions (including assessment of possible causes)	Supports intensive therapy and allo-HSCT in eligible patients
	11d	The primary "take-away" lessons of this case report (without references) in a one paragraph conclusion	Early recognition of atypical skin lesions is crucial
Patient Perspective	12	The patient should share their perspective in one to two paragraphs on the treatment(s) they received	Patient initially underestimated lesions and was satisfied with treatment outcome
Informed Consent	13	Did the patient give informed consent? Please provide if requested	Yes ☒ No ☐
